# Development and External Validation of an Explainable AHP-ML Model for Orthodontic Tooth Extraction and Anchorage Decision Support

**DOI:** 10.3390/bioengineering13060671

**Published:** 2026-06-10

**Authors:** Yang Yi, Xinhang Shen, Bin Wu, Yingyu Chen, Mao Liu, Bin Yan

**Affiliations:** 1 College of Mechanical and Electronic Engineering, Nanjing Forestry University, Nanjing 210037, China; 2 Department of Orthodontics, School of Stomatology, Nanjing Medical University, Nanjing 210029, China; 3 Jiangsu Key Laboratory of Oral Diseases, Nanjing Medical University, Nanjing 210029, China; 4 Jiangsu Key Laboratory for Design and Manufacture of Micro-Nano Biomedical Instruments, School of Mechanical Engineering, Southeast University, Nanjing 211000, China

**Keywords:** orthodontic treatment decision support, tooth extraction, anchorage control, Analytic Hierarchy Process, explainable machine learning

## Abstract

Tooth extraction and maximum anchorage assessment are key decision points in orthodontic treatment planning, yet existing machine learning models for orthodontic decision support often lack transparency, limiting their clinical interpretability and trustworthiness. In this study, we developed and externally validated an explainable orthodontic treatment decision-support model that integrates expert-derived Analytic Hierarchy Process (AHP) weighting with machine learning. A diagnostic indicator framework comprising 18 orthodontic variables was established through a literature review, clinical data analysis, and two rounds of expert surveys. A retrospective cohort of 485 patients receiving fixed-appliance orthodontic treatment was used for model development and internal validation. AHP-derived composite scores were incorporated into the machine learning models for two prediction tasks, namely tooth extraction and maximum anchorage requirement, and an expert-informed fuzzy-rule score was calculated from pretreatment indicators for the maximum anchorage task to capture clinically interpretable anchorage tendencies. Model performance was evaluated using ROC-AUC, F1 score, precision, recall, PR-AUC, calibration analysis, and decision curve analysis, while SHAP was applied to interpret feature contributions. The AHP-RF extraction model and AHP-enhanced LR maximum anchorage model achieved the highest AUCs among the compared models (0.864 and 0.822, respectively), although paired DeLong tests showed no significant differences from the closest competing models. SHAP analysis identified lower lip-to-E-line distance, U1-NA, and the AHP composite score as important predictors, indicating consistency between model outputs and clinical reasoning. In the external validation cohort, the extraction model correctly classified 57 of 74 cases, and the maximum anchorage model correctly classified 24 of 29 cases, supporting the preliminary transportability of the proposed framework. These results suggest that integrating AHP-derived expert knowledge with machine learning provides an explainable and clinically interpretable decision-support model for orthodontic treatment planning, with potential value in improving standardized, evidence-informed, and patient-specific orthodontic decision-making.

## 1. Introduction

Orthodontic diagnostic assessment and treatment planning involve multiple complex clinical decision points, among which tooth extraction and anchorage control are of central importance. These decisions directly influence soft-tissue esthetics, occlusal function, and the long-term stability of treatment outcomes [1,2]. Traditionally, orthodontic treatment planning has relied heavily on clinicians’ subjective experience, resulting in variability among practitioners and a lack of standardized, reproducible criteria.

**Figure 1:**
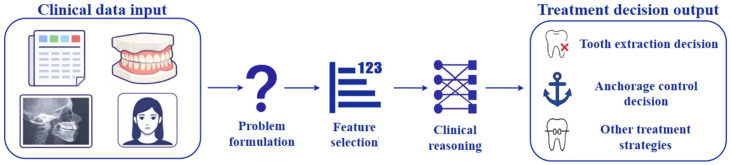
Schematic representation of the orthodontic decision-making process.

Figure 1 summarizes the clinical workflow underlying orthodontic treatment decision-making. In routine practice, clinicians integrate patient information, chief complaints, dental casts, cephalometric measurements, and soft-tissue assessments to determine individualized treatment strategies. Among these decisions, extraction assessment and anchorage planning are particularly critical because they influence space management, anterior tooth retraction, facial profile improvement, and occlusal stability. Extraction cases may require varying degrees of anchorage control during space closure, whereas non-extraction approaches generally aim to preserve arch form and facial harmony. This clinical rationale is consistent with the classical orthodontic principles established by Graber et al. [3] and Sarver [4].

With the rapid advancement of artificial intelligence (AI), machine learning (ML) has demonstrated strong potential for pattern recognition and clinical prediction in medicine [5,6]. In orthodontics, several studies have applied ML to treatment planning tasks, such as extraction decisions, combined orthodontic-orthognathic treatment recommendations, and tooth movement simulation [7,8]. In recent years, AI has been increasingly recognized as a transformative force in orthodontics, offering new opportunities for data-driven diagnosis and treatment planning [9]. Li et al. [10] developed an extraction prediction model using artificial neural networks, demonstrating improved accuracy but limited by a small sample size and a lack of external validation. Prasad et al. [11] proposed a clinical decision-support system based on ML, while Mason et al. [12] incorporated multimodal features to enhance the prediction of extraction and anchorage decisions. More broadly, recent AI applications in orthodontics have extended from treatment-decision prediction to cephalometric analysis and maxillofacial morphology classification, supporting more standardized and data-driven diagnostic and treatment planning workflows [13,14]. Although these studies highlight the feasibility of ML in orthodontic decision-making, many existing models remain black-box approaches, providing limited interpretability and reducing clinicians’ trust in model outputs.

To address this challenge, integrating expert knowledge with data-driven models has emerged as a promising approach to enhance transparency and reliability. The Analytic Hierarchy Process (AHP), a structured multi-criteria decision-making method, systematically incorporates expert judgment to assign relative importance to diagnostic indicators [15,16,17]. When combined with ML and explainable algorithms such as SHAP [18,19], AHP can bridge the gap between clinical reasoning and predictive analytics, creating models that are both accurate and interpretable. Accordingly, this study aimed to develop and externally evaluate an explainable orthodontic treatment decision-support framework that integrates AHP-derived expert weighting with machine learning. The framework focused on two clinically important treatment planning tasks: tooth extraction assessment and maximum anchorage requirement prediction.

The main contributions of this study are: (i) AHP-derived expert weights were converted into a composite score and incorporated as an additional ML feature; (ii) SHAP analysis was used to identify key predictors and interpret model outputs; (iii) the model first predicted tooth extraction and then assessed maximum anchorage among extraction cases.

## 2. Materials and Methods

### 2.1. Study Design and Patient Cohorts

**Figure 2:**
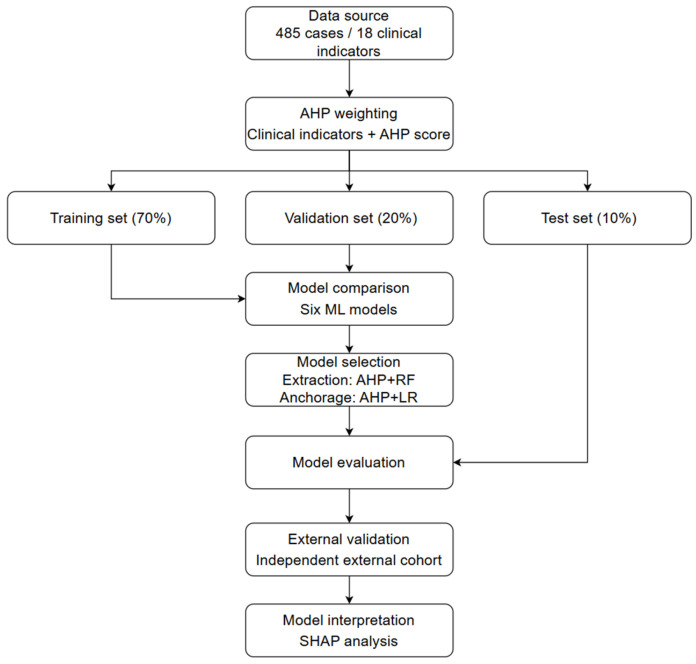
Workflow of the AHP-ML-based orthodontic decision-support framework.

This retrospective study used a model-development and external-validation design, as illustrated in Figure 2. The internal development cohort included 485 patients who received fixed-appliance orthodontic treatment at Nanjing Medical University between 2017 and 2024. After model development and internal model selection were completed, an independent external validation cohort was retrospectively assembled from eligible patients treated at Tianjin Stomatological Hospital between 2024 and 2025. The external cohort was not used for feature selection, model training, hyperparameter tuning, or threshold selection. Therefore, the two cohorts overlapped in 2024, with no temporal gap, while the external cohort additionally included cases treated in 2025.

Patients were included if they met the following criteria: (1) permanent dentition; (2) initial age between 10 and 45 years; and (3) availability of complete clinical and radiographic records required for extracting pretreatment variables and historical treatment planning labels. Exclusion criteria were: (1) incomplete clinical or radiographic data, including missing values in variables required for analysis; (2) insufficient documentation to determine the historical extraction or anchorage treatment planning labels; (3) a history of bone-related diseases or long-term medication use affecting bone metabolism; and (4) previous orthodontic treatment. Cases with missing values in variables required for analysis were excluded during dataset assembly; therefore, the final analytic dataset contained complete records only, and no imputation was performed.

The final internal development cohort consisted of 485 orthodontic patients, from whom 18 pretreatment clinical indicators were extracted for model construction.

### 2.2. Clinical Indicators

Baseline diagnostic data were collected from pretreatment radiographs and dental models. A total of 18 pretreatment clinical indicators were selected based on prior literature, routine orthodontic diagnostic frameworks, data completeness, and clinical relevance to extraction and maximum anchorage decisions. The indicator set was further reviewed and confirmed by an expert panel before AHP weighting.

The selected indicators were grouped into three categories. Demographic variables included age (years) and sex. Dental model variables included molar relationship, overbite, overjet, upper arch crowding (mm), lower arch crowding (mm), and curve of Spee (mm). Cephalometric variables included ANS-Me (mm), facial convexity angle (°), lower lip-to-E-line distance (mm), nasolabial angle (°), U1-NA (°), L1-NB (°), ANB (°), SNA (°), SNB (°), and MP-FH (°). These variables collectively reflect skeletal, dental, and soft-tissue characteristics and provided the basis for constructing task-specific feature sets for the extraction and maximum anchorage prediction models.

To assess the reliability of cephalometric measurements, two observers independently repeated the measurements in a subset of 20 cases. Inter-observer reliability was evaluated using the ICC and Dahlberg error. Overall, the measurements showed excellent agreement between observers, with ICC values ranging from 0.907 to 0.998 and acceptable Dahlberg errors. The detailed results are presented in Supplementary Table S1.

### 2.3. AHP-Based Expert Knowledge Integration

To incorporate expert knowledge into the prediction framework, AHP was used to derive relative weights for the diagnostic indicators. Two rounds of structured surveys were conducted among 10 experienced orthodontic specialists, including chief and associate chief physicians, each with at least 5 years of clinical experience. Experts completed the pairwise comparisons independently, and individual judgments were aggregated using the geometric mean to construct a group pairwise comparison matrix.

In the AHP procedure, a pairwise comparison matrix $A=[a_{ij}{]}_{n\times n}\;$was constructed, where each element $a_{ij}$ represents the relative importance of indicator i compared with indicator j on a 1–9 scale. The normalized principal eigenvector w of the pairwise comparison matrix A yields the weight vector, satisfying $Aw=\lambda_{\max}w$. The consistency of expert judgments was verified using the Consistency Ratio (*CR*), defined as:

$$CR=\frac{CI}{RI}$$

$$CI=\frac{\lambda_{max}-n}{n-1}$$

A decision matrix was accepted only if CR<0.1, indicating acceptable logical consistency. The final group matrices showed acceptable consistency for both decision tasks: extraction decision, $\lambda_{max}$ = 11.327, CI = 0.033, RI = 1.51, CR = 0.022; maximum anchorage decision, $\lambda_{max}$ = 9.248, CI = 0.031, RI = 1.45, CR = 0.021.

For each patient, an AHP composite score $S_{i}\;$was computed as the weighted sum of all standardized diagnostic indicators:

$$S_{i}=\sum_{k=1}^{n}w_{k}x_{ik}$$

$$z_{i}=\left[x_{i1},x_{i2},\ldots ,x_{in},S_{i}\right]$$

where $x_{ik}\;$ represents the normalized value of indicator k  for patient i, and $w_{k}\;$ denotes the expert-derived weight. This score represented an expert-informed quantitative assessment and was incorporated into the feature set for model training, where $z_{i}$ denotes the final input feature vector used for machine learning model training. The indicator rankings and corresponding AHP-derived weights for the extraction and maximum anchorage tasks are provided in Table 1.

### 2.4. Development of the Explainable AHP-ML Model

Following the AHP-based expert knowledge integration described above, an explainable AHP-ML framework was developed to support two key orthodontic decisions: tooth extraction and maximum anchorage requirement. The AHP composite score was incorporated as an additional expert-informed feature together with the standardized clinical indicators, allowing expert-derived clinical reasoning to be embedded into the machine learning input space. For the anchorage-control task, an additional expert-derived fuzzy comprehensive score was further generated based on clinically defined anchorage-related rules and expert scoring and was included as a supplementary knowledge-based feature to reflect the tendency toward maximum anchorage in borderline or clinically complex cases. The detailed scoring criteria for this expert-informed fuzzy-rule score are presented in Supplementary Table S2. The score was calculated only from pretreatment anchorage-related indicators and was used as an engineered input feature rather than as a rule for modifying historical anchorage labels. The thresholds were tuned within the training set and then fixed before validation, internal testing, and external validation to reduce the risk of information leakage.

The framework followed a two-stage decision process consistent with the clinical orthodontic workflow. In the first stage, all eligible cases were used to develop the tooth extraction prediction model. In the second stage, a separate anchorage-control model was developed only for extraction cases to determine whether maximum or reinforced anchorage was required. In this study, maximum anchorage was operationally defined as the treatment objective of preserving posterior anchorage and minimizing mesial movement of the posterior teeth during space closure, regardless of whether this was achieved using skeletal anchorage or reinforced conventional appliances [20]. Therefore, the maximum anchorage label should be interpreted as an inclusive indication for any reinforced anchorage requirement, rather than as a prediction of a specific anchorage appliance or construct. Cases requiring only reciprocal, moderate, or routine anchorage control were classified as conventional anchorage.

Several ML algorithms were developed and compared for the two prediction tasks, including two AHP-enhanced models, namely AHP+RF and AHP+LR, and four conventional ML models, including Original RF, XGBoost, SVM, and GBDT. RF was primarily used for extraction prediction because of its ability to capture nonlinear relationships and interactions among multiple clinical indicators. LR was adopted as the main model for anchorage control prediction because of its probabilistic output and relatively transparent decision structure. Similar multi-algorithm comparisons were reported by Köktürk et al. [21], who evaluated various machine learning models for orthodontic extraction decisions.

Continuous variables were normalized using training-set-based min-max scaling. Categorical variables were numerically encoded before AHP score calculation and model training. Molar relationship was coded as Class I = 0, Class II = 1, and Class III = 2. Overbite was coded as anterior open bite = −1, normal = 0, mild deep overbite = 1, moderate deep overbite = 2, and severe deep overbite = 3. Overjet was coded as reverse overjet = −1, normal = 0, mild increased overjet = 1, moderate increased overjet = 2, and severe increased overjet = 3. The internal cohort was then randomly divided into training, validation, and held-out test sets at a ratio of 7:2:1. The training and validation sets were used for model fitting, hyperparameter tuning, and model selection, whereas the held-out test set was reserved for final internal performance assessment. Hyperparameters were optimized using grid search with five-fold cross-validation, and the final settings were selected according to the highest mean AUC across the validation folds.

### 2.5. Model Evaluation and External Validation

Model performance was assessed on the held-out internal test set after model development and hyperparameter optimization. Predicted probabilities were converted into binary recommendations using a pre-specified threshold of 0.50 for both prediction tasks, with probabilities ≥ 0.50 indicating a positive prediction. This threshold was used as a default cut-off for consistent comparison across tasks and algorithms rather than as a clinically optimized decision threshold. To examine the robustness of threshold-dependent performance, additional threshold-sensitivity analyses using the Youden-index threshold and the maximum-net-benefit threshold were performed for the selected models, as reported in Supplementary Table S3. For extraction prediction, a positive prediction indicated tooth extraction, whereas for anchorage control prediction, a positive prediction indicated the requirement for maximum anchorage.

The discriminative performance of each model was evaluated using the area under the receiver operating characteristic curve (AUC). Classification performance was further assessed using the F1 score, precision, and recall. Considering the moderate class imbalance in the anchorage-control task, the area under the precision–recall curve (PR-AUC) was additionally reported as a complementary metric. Because PR-AUC is prevalence-dependent, it was interpreted relative to the no-skill baseline defined by the positive-class prevalence rather than as a standalone primary measure of model superiority.

Model calibration was assessed using pooled five-fold calibration curves and Brier scores, which were used to evaluate the agreement between predicted probabilities and observed outcomes. Decision curve analysis (DCA) was performed to assess the potential clinical utility of each model across a range of threshold probabilities by estimating the net benefit under different decision thresholds.

SHAP analysis was performed on the selected models to examine model interpretability. Global feature importance was quantified using mean absolute SHAP values, whereas SHAP beeswarm plots were used to visualize both the direction and distribution of feature effects.

External validation was conducted to evaluate the transportability of the proposed framework beyond the development center. The two selected internally validated models were further assessed using an independent cohort of 74 cases from Tianjin Stomatological Hospital. These external cases were completely excluded from model training, hyperparameter optimization, model selection, and internal testing.

### 2.6. Statistical Analysis

All statistical analyses were conducted using R (version 4.2.3) and Python (version 3.11). Continuous variables were expressed as mean ± standard deviation (SD) and compared using Welch’s t-test. Categorical variables were presented as frequencies and percentages and compared using the χ^2^ test or Fisher’s exact test, as appropriate. All tests were two-sided, and *p* < 0.05 was considered statistically significant. The baseline comparisons in Table 2 and Table 3 were performed for descriptive characterization of the cohort only and were not used for inferential feature selection or model optimization; therefore, the reported *p* values were not adjusted for multiple comparisons.

## 3. Results

### 3.1. Baseline Characteristics

A total of 485 patients met the inclusion criteria and were analyzed. As shown in Table 2, compared with the non-extraction group, the extraction group showed greater upper and lower arch crowding, more pronounced incisor proclination (U1-NA and L1-NB), greater lower lip-to-E-line distances, and smaller nasolabial angles (all *p* < 0.001). Differences in SNB and MP-FH (Frankfort mandibular plane angle, FMA) were also observed between the two groups.

For anchorage control (i.e., the requirement for maximum anchorage), as summarized in Table 3, significant between-group differences were observed in age, molar relationship, overbite, overjet, lower lip-to-E-line distance, U1-NA, and L1-NB. ANS-Me was not statistically significant after recalculation and was therefore no longer described as a significant variable. Although age showed a statistically significant difference, it was not included in the final anchorage-control model because maximum anchorage requirement is clinically determined mainly by dental, soft-tissue, and biomechanical considerations rather than by age alone. Variables without statistical significance in univariate comparisons, such as ANS-Me and arch crowding, were retained when they were considered clinically relevant by the expert panel and routinely used in anchorage planning.

Based on the initial 18-indicator clinical framework, task-specific raw input sets were determined before model development. This reduction was based on prior orthodontic literature, clinical relevance to each decision task, data completeness, and expert-panel review, rather than recursive feature elimination, model-based selection, or optimization on the held-out test set. The selected feature sets were fixed before model training and were applied consistently across the train–test split and cross-validation folds. Accordingly, the final extraction–prediction model incorporated 11 raw clinical indicators, including upper and lower arch crowding, U1-NA, L1-NB, lower lip-to-E-line distance, nasolabial angle, SNB, SNA, ANB, curve of Spee, and MP-FH, together with the AHP composite score. In contrast, the anchorage-control model retained 9 raw clinical indicators, including molar relationship, overbite, overjet, upper and lower arch crowding, U1-NA, L1-NB, lower lip-to-E-line distance, and ANS-Me, together with the AHP composite score.

### 3.2. Predictive Performance of the AHP-ML Models

**Figure 3:**
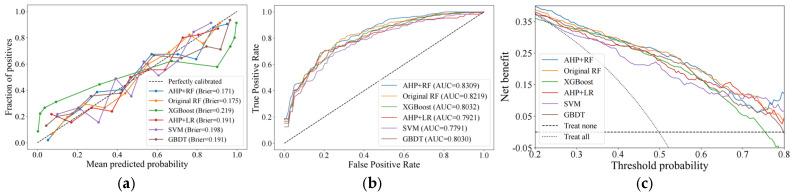
Performance comparison of six models for extraction prediction. (**a**) Calibration curves (5-fold pooled) with Brier scores; (**b**) five-fold cross-validation ROC curves; (**c**) Decision curve analysis (DCA).

As illustrated in Figure 3 and Table 4, the AHP+RF model exhibited reasonable calibration and achieved the lowest Brier score (0.171), compared with the Original RF model (0.175) and other baseline models, indicating relatively better probability estimation. Discrimination and stability were then evaluated using five-fold cross-validation ROC curves, where AHP+RF performed among the best models, with a mean AUC of 0.831. To further examine the incremental contribution of the AHP composite score in the extraction task, we added an ablation analysis comparing the clinical-indicator-only RF model with the RF model incorporating both clinical indicators and the AHP composite score. As shown in Supplementary Table S4a, adding the AHP composite score increased the AUC from 0.820 to 0.864 and slightly reduced the Brier score from 0.175 to 0.171. Finally, decision curve analysis suggested that AHP+RF provided a higher net clinical benefit than alternative models across a broad range of threshold probabilities, supporting its potential clinical utility.

**Figure 4:**
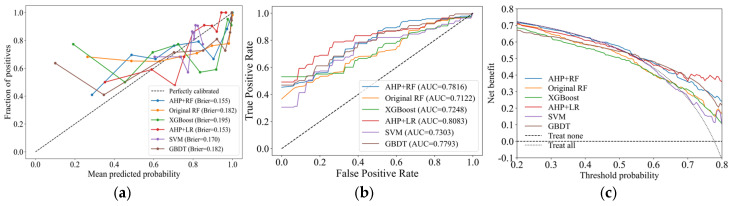
Performance comparison of six models for anchorage control prediction. (**a**) Calibration curves (5-fold pooled) with Brier scores; (**b**) five-fold cross-validation ROC curves; (**c**) DCA.

For maximum anchorage prediction, the positive class accounted for 63.6% of cases, indicating a moderately imbalanced setting. PR-AUC was therefore reported as a complementary metric and interpreted relative to the prevalence-based no-skill baseline of 0.636. As shown in Figure 4 and Table 5, the AHP+LR model showed the highest AUC point estimate (AUC = 0.822), the highest PR-AUC (0.934), and the highest F1 score (0.941) at the pre-specified threshold. Calibration assessment using 5-fold pooled calibration curves further supported its reliability, yielding the lowest Brier score (0.153) compared with those of other models. Decision curve analysis indicated that AHP+LR provided a higher net benefit than alternative models across a broad range of clinically relevant thresholds. In contrast, AHP+RF showed comparatively lower discrimination (AUC = 0.808), suggesting that a parsimonious and interpretable linear model may better capture the decision pattern for maximum anchorage assessment in this dataset. As shown in Supplementary Table S4b, the final model incorporated both the AHP composite score and the expert-informed fuzzy-rule score. Compared with the clinical-indicator-only LR model, it improved the AUC from 0.806 to 0.822 and reduced the Brier score from 0.172 to 0.153. However, adding the fuzzy-rule score alone increased the AUC only from 0.806 to 0.808, suggesting that this score should be interpreted mainly as a clinically interpretable aggregated proxy feature rather than an independent source of substantial new predictive information. Paired DeLong tests were further performed to compare the AUCs of the selected models with those of alternative models, and the results are reported in Supplementary Table S5. Paired DeLong tests indicated that the selected models had the highest AUC point estimates but were not statistically superior to their nearest competitors. AHP+RF was not significantly different from XGBoost (*p* = 0.062) or GBDT (*p* = 0.053) for extraction prediction, and AHP+LR was not significantly different from AHP+RF (*p* = 0.081) or SVM (*p* = 0.073) for maximum anchorage prediction.

**Figure 5:**
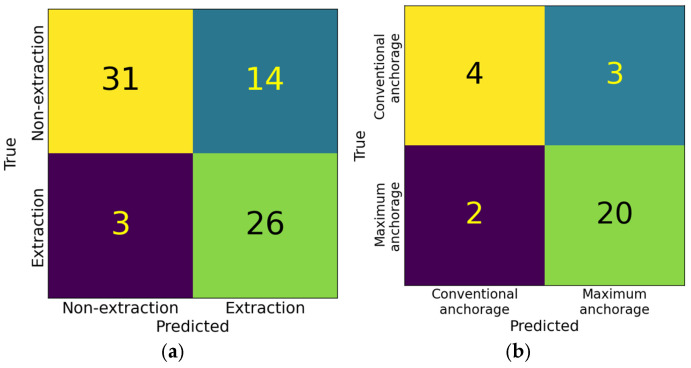
Confusion matrices of the two selected models in the external validation set. (**a**) Extraction prediction by AHP+RF; (**b**) anchorage prediction by AHP+LR.

To further assess model transportability beyond the development center, the two selected internally validated models were evaluated in an independent external validation cohort. Figure 5 presents the corresponding confusion matrices. In the external cohort, the AHP+RF extraction model was evaluated in all 74 cases and achieved 57/74 correct classifications (77.0%). The AHP+LR maximum anchorage model was first evaluated conditionally in the 29 cases that had undergone tooth extraction according to the historical treatment records, achieving 24/29 correct classifications (82.8%). We also added an exploratory end-to-end cascade analysis to reflect the deployable workflow. After first-stage extraction prediction, 40 of 74 external patients entered the second-stage anchorage model, while three true extraction cases were missed by the first stage. Among the true extraction cases, the final downstream error count was six. The full cascade results are reported in Supplementary Table S6. This conditional result represents second-stage model validation under the true historical extraction condition rather than full end-to-end deployment performance. Additional external-validation performance metrics are summarized in Supplementary Table S7. The calibration intercepts were −0.271 for the extraction model and −0.316 for the maximum anchorage model, suggesting mild overall overestimation of the positive-class probabilities. The wide confidence intervals for the maximum anchorage model, especially for AUC and specificity, indicate that the external validation results should be interpreted cautiously. External calibration and decision-curve analyses are shown in Supplementary Figure S1, and baseline comparisons between the development and external validation cohorts are provided in Supplementary Table S8a,b.

### 3.3. Feature Importance and Explainability

SHAP analysis was performed on the held-out test set for both prediction tasks to interpret the selected models. SHAP values quantify the magnitude and direction of each feature’s contribution to model predictions, thereby improving model transparency and facilitating comparison with clinical reasoning.

**Figure 6:**
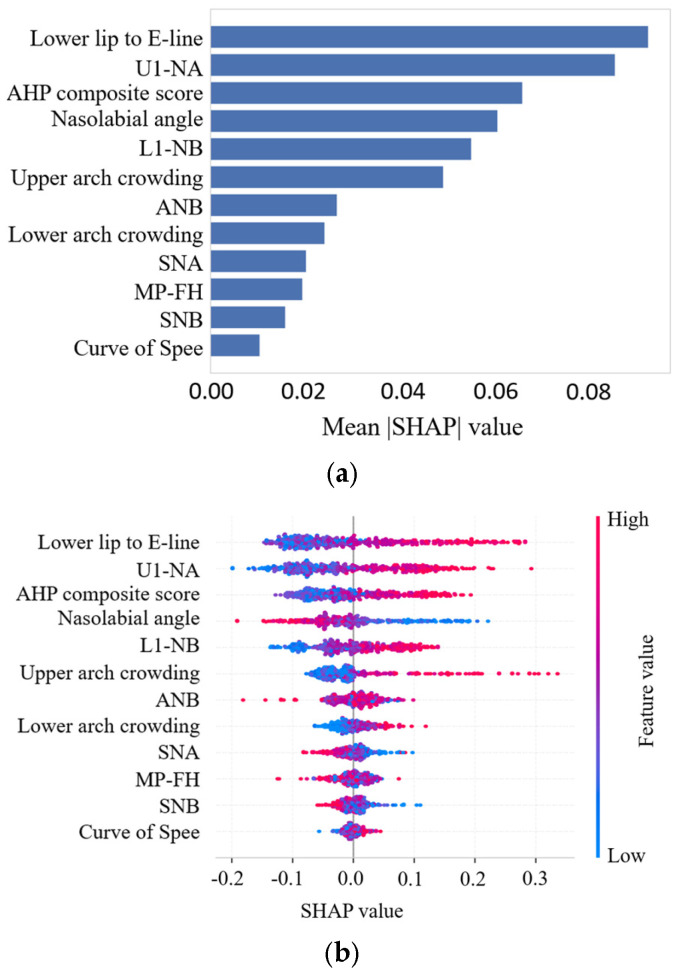
SHAP explainability analysis for the extraction model on the held-out test set. (**a**) SHAP summary bar plot. (**b**) SHAP beeswarm plot.

As illustrated in Figure 6, for the extraction model, the most influential predictors were lower lip-to-E-line distance, U1-NA, and the AHP composite score. These variables contributed most strongly to the model output and were consistent with established clinical considerations in extraction decision-making. In particular, the presence of the AHP composite score among the top-ranked features indicates that expert-derived weighting provided additional predictive information beyond individual clinical measurements. The SHAP beeswarm plot further showed that greater incisor proclination and lip protrusion generally tended to increase the predicted probability of extraction.

**Figure 7:**
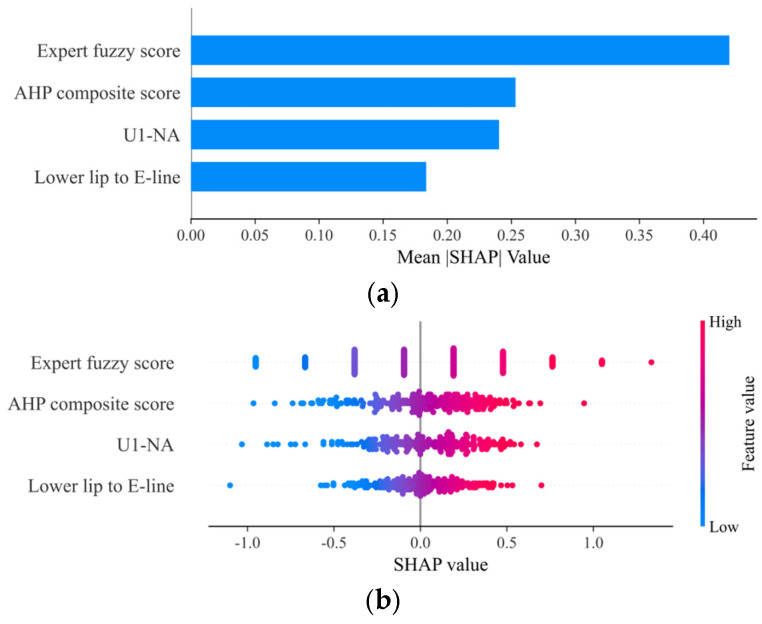
SHAP explainability analysis for the maximum anchorage model on the held-out test set. (**a**) SHAP summary bar plot. (**b**) SHAP beeswarm plot.

For the maximum anchorage model, Figure 7 shows that the expert-informed fuzzy-rule score has the largest mean absolute SHAP value, followed by the AHP composite score, U1-NA, and lower lip-to-E-line distance. However, because the fuzzy-rule score is derived from pretreatment clinical indicators that are also included in the model, this high SHAP attribution should be interpreted cautiously. It likely reflects attribution redistribution among correlated and redundant features, rather than a large independent increase in predictive discrimination. Therefore, the fuzzy-rule score is best understood as an aggregated, clinically interpretable proxy feature that summarizes several anchorage-related tendencies already present in the input space. The beeswarm plot indicates that higher values of the expert-informed features, together with greater incisor proclination and lip protrusion, generally contribute to a higher predicted probability of maximum anchorage.

### 3.4. Preliminary Clinician Feedback

To obtain preliminary user feedback on the prototype system, a decision-support system based on the proposed models was developed and reviewed by a panel of five experienced orthodontists. The five clinicians who participated in this evaluation partially overlapped with the AHP expert panel. Therefore, this section should be interpreted as preliminary user feedback rather than independent clinical validation. Ten representative cases with varying malocclusion types and treatment complexities were selected for review. The model-generated recommendations were compared descriptively with the actual historical treatment plans.

As summarized in Table 6, experts rated the system highly across four dimensions—diagnostic accuracy, decision-making efficiency, interpretability, and overall clinical value—with average scores ranging from 8.5 to 9.3 out of 10. Representative feedback indicated that the system closely mirrors clinicians’ reasoning and helps reduce inter-practitioner variability, supporting both clinical decision-making and educational training. Importantly, this feedback highlights the incremental value of a measurement-only approach: by standardizing the decision logic and providing instant recommendations, the system can improve consistency across clinicians and streamline the initial planning workflow without requiring additional imaging acquisition or manual landmarking.

## 4. Discussion

A major contribution of this study lies in the use of AHP not only as a weighting method but also as a mechanism for embedding expert knowledge into ML models. This design enabled the models to learn from both original clinical indicators and an aggregated expert-informed assessment. Compared with conventional models using original indicators alone, the AHP-enhanced models showed improved predictive performance, suggesting that the AHP composite score provided complementary information for orthodontic decision-making. SHAP analysis showed that the AHP composite score ranked among the most influential features alongside established clinical parameters, supporting the conclusion that expert knowledge contributed to transparent decision logic and improved clinical interpretability.

In the extraction prediction task, dental crowding and incisor proclination emerged as the most decisive predictors, consistent with established orthodontic principles that identify tooth size-arch length discrepancy and anterior protrusion as primary indications for extraction [1,2,3]. These findings also align with previous studies demonstrating that extraction can optimize lip–tooth relationships and facial esthetics [22,23]. Soft-tissue indicators, such as the lower lip-to-E-line distance and nasolabial angle, were also influential, supporting Sarver’s soft-tissue-oriented treatment philosophy [4]. For anchorage control, U1-NA and the lower lip-to-E-line distance were identified as the strongest predictors, corroborating the findings of Xu et al. [24] and Li et al. [10], who emphasized the role of incisor inclination and lip position in determining anchorage requirements. These results suggest that both hard- and soft-tissue parameters are essential for reliable anchorage planning, a principle also supported by Liu et al. [25].

**Figure 8:**
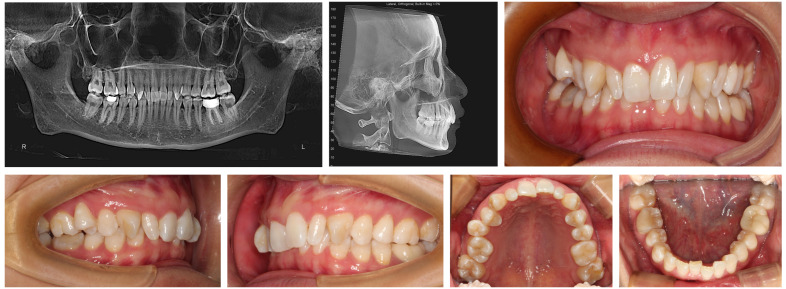
Representative atypical case with pseudo-enlarged ANB caused by dentoalveolar compensation.

Several limitations of this study should be acknowledged. First, the model was developed mainly using a single-center retrospective cohort, and the held-out internal test sets were relatively small. Therefore, model comparisons should be interpreted cautiously, and the observed differences should not be regarded as definitive evidence of model superiority. Although an independent external validation cohort was included, its sample size remained limited. Moreover, the external maximum anchorage evaluation was primarily a conditional validation of the second-stage model based on true historical extraction cases, rather than a full end-to-end assessment of the deployed cascaded workflow. In such a workflow, errors from the first-stage extraction model may propagate to the anchorage-control stage because cases incorrectly classified as non-extraction would not enter the second-stage anchorage module. Accordingly, larger multicenter studies and prospective end-to-end evaluations are needed to further assess model generalizability and real-world clinical applicability. Second, atypical cases with abnormal incisor position or functional malocclusion may affect cephalometric measurements such as ANB, as illustrated in Figure 8, potentially leading to false-positive extraction predictions [26]. Future work will consider incorporating additional sagittal indices less dependent on point A, such as the Wits appraisal, Beta angle, and APDI, to improve robustness in such cases. Third, patients requiring combined orthodontic-orthognathic treatment were not included, and functional factors were only indirectly considered. Future studies should extend the framework to these populations while also integrating radiographs, intraoral images, 3D scans, and functional assessment data to capture richer morphological information. Fourth, the signed ordinal encoding of overbite and overjet and the intentionally inclusive definition of maximum anchorage should be considered additional limitations. Future model updates should consider one-hot or direction-and-severity encoding for occlusal variables, and the anchorage model should be further validated in centers with different anchorage practice patterns or appliance preferences. Fifth, intra-observer repeat measurements and repeated dental model measurements were unavailable because of the retrospective study design. Therefore, reliability estimates for dental model variables, including arch crowding, curve of Spee, overbite, and overjet, could not be reported, which may introduce additional measurement uncertainty.

In summary, the integration of AHP-derived expert knowledge with machine learning provides a preliminary and interpretable framework for modeling historical orthodontic treatment planning decisions. This hybrid approach improves model performance, enhances transparency, and aligns algorithmic prediction with clinical reasoning. The proposed decision-support model holds promise for standardizing orthodontic treatment planning, reducing inter-practitioner variability, and facilitating evidence-based, patient-specific care.

## 5. Conclusions

This study developed an explainable, expert-augmented decision-support framework for orthodontic treatment planning by integrating AHP weighting with machine learning. By combining expert-derived knowledge with data-driven prediction, the proposed AHP-ML models demonstrated promising performance and improved interpretability for both extraction and maximum anchorage decision support. SHAP-based explanations further enhanced transparency by providing feature-level contributions that clinicians can review alongside established clinical reasoning.

Future work will focus on larger multicenter and prospective validation studies to assess model generalizability across different populations and clinical settings. In addition, integrating radiographs, intraoral images, and 3D scans may capture richer morphological information and improve model robustness. After adequate validation, the proposed framework may serve as an auxiliary clinical software tool to support more transparent and consistent orthodontic treatment planning discussions.

## Figures and Tables

**Table 1:** AHP-derived indicator ranks and weights for extraction and anchorage tasks.

Clinical Indicator	Extraction Rank	Extraction Weight	Anchorage Rank	Anchorage Weight
Lower lip-to-E-line distance (mm)	1	0.108	2	0.160
Upper arch crowding (mm)	2	0.104	3	0.120
Lower arch crowding (mm)	3	0.098	6	0.090
Nasolabial angle (°)	4	0.097	-	-
ANB (°)	5	0.095	-	-
U1-NA (°)	6	0.088	1	0.180
L1-NB (°)	7	0.084	5	0.100
SNA (°)	8	0.084	-	-
MP-FH (°)	9	0.084	-	-
Curve of Spee (mm)	10	0.081	-	-
SNB (°)	11	0.077	-	-
molar relationship	-	-	4	0.110
Overbite	-	-	7	0.080
Overjet	-	-	8	0.080
ANS-Me (mm)	-	-	9	0.080

**Table 2:** Comparison of baseline clinical characteristics between extraction and non-extraction groups.

Variable	Total (n = 485)	Non-Extraction Group (n = 246)	Extraction Group (n = 239)	*p* Value
Age (years)	17.58 ± 6.82	17.54 ± 7.14	17.62 ± 6.51	0.917
Sex				0.523
Male	164	85 (34.6%)	79 (33.1%)	
Female	321	161 (65.4%)	160 (66.9%)	
Molar relationship				0.315
Class I	212	110 (44.7%)	102 (42.7%)	
Class II	202	102 (41.5%)	100 (41.8%)	
Class III	71	34 (13.8%)	37 (15.5%)	
Overbite				0.344
Normal	162	77 (31.3%)	85 (35.6%)	
Mild	65	38 (15.4%)	27 (11.3%)	
Moderate	99	52 (21.1%)	47 (19.7%)	
Severe	128	60 (24.4%)	68 (28.5%)	
Anterior open bite	31	19 (7.7%)	12 (5.0%)	
Overjet				0.074
Normal	170	88 (35.6%)	82 (34.2%)	
Mild	131	78 (31.6%)	53 (22.1%)	
Moderate	99	45 (18.2%)	54 (22.5%)	
Severe	54	18 (7.3%)	36 (15.0%)	
Reverse overjet	31	18 (7.3%)	15 (6.3%)	
ANS-Me (mm)	66.17 ± 7.48	65.71 ± 7.37	66.62 ± 7.58	0.279
Facial convexity angle (°)	12.60 ± 5.92	12.39 ± 5.61	12.81 ± 6.21	0.673
Lower lip-to-E-line (mm)	1.95 ± 3.10	0.93 ± 2.62	3.0 ± 3.21	<0.001
Nasolabial angle (°)	102.96 ± 10.03	105.2 ± 9.41	100.65 ± 10.15	<0.001
Upper arch crowding (mm)	3.25 ± 3.68	2.5 ± 3.13	4.02 ± 4.03	<0.001
Lower arch crowding (mm)	2.73 ± 3.26	2.17 ± 3.03	3.32 ± 3.39	<0.001
U1-NA (°)	25.97 ± 9.14	23.49 ± 8.64	28.52 ± 8.96	<0.001
L1-NB (°)	27.66 ± 6.89	25.63 ± 6.46	29.75 ± 6.71	<0.001
ANB (°)	3.87 ± 2.65	3.66 ± 2.77	4.08 ± 2.5	0.086
SNA (°)	81.76 ± 3.73	81.86 ± 3.7	81.66 ± 3.76	0.562
SNB (°)	77.76 ± 5.12	78.22 ± 4.15	77.3 ± 5.93	0.047
Curve of Spee (mm)	2.88 ± 1.23	2.82 ± 1.21	2.95 ± 1.26	0.233
MP-FH (°)	25.83 ± 6.63	25.08 ± 6.15	26.6 ± 7.01	0.011

**Table 3:** Comparison of baseline clinical characteristics between maximum anchorage and conventional anchorage groups.

Variable	Total (n = 239)	Conventional Anchorage Group	Maximum Anchorage Group	*p* Value
Age (years)	16.96 ± 6.79	17.82 ± 6.12	15.84 ± 6.60	0.020
Sex				0.073
Male	75	34 (39.1%)	41 (27.0%)	
Female	164	53 (60.9%)	111 (73.0%)	
Molar relationship				**<0.001**
Class I	122	19 (21.8%)	103 (67.8%)	
Class II	77	41 (47.1%)	36 (23.7%)	
Class III	40	27 (31.0%)	13 (8.6%)	
Overbite				**0.003**
Normal	93	23 (25.5%)	70 (47.0%)	
Mild	29	14 (15.6%)	15 (10.1%)	
Moderate	54	15 (16.6%)	39 (26.2%)	
Severe	39	16 (17.8%)	23 (15.4%)	
Anterior open bite	24	22 (24.4%)	2 (1.3%)	
Overjet				**0.043**
Normal	98	32 (36.8%)	66 (43.4%)	
Mild	46	18 (20.7%)	28 (18.4%)	
Moderate	42	18 (20.7%)	24 (15.8%)	
Severe	29	3 (3.4%)	26 (17.1%)	
Reverse overjet	24	16 (18.4%)	8 (5.3%)	
ANS-Me (mm)	66.82 ± 6.79	67.70 ± 5.33	66.31 ± 7.46	0.096
Facial convexity angle (°)	13.22 ± 6.67	12.13 ± 6.16	13.94 ± 6.89	0.216
Lower lip-to-E-line (mm)	3.00 ± 3.24	2.08 ± 2.61	3.44 ± 3.42	**0.001**
Nasolabial angle (°)	100.74 ± 10.19	101.23 ± 9.61	100.50 ± 10.48	0.601
Upper arch crowding (mm)	4.08 ± 4.06	4.25 ± 3.96	4.00 ± 4.11	0.65
Lower arch crowding (mm)	3.34 ± 3.42	3.43 ± 3.44	3.30 ± 3.41	0.787
U1-NA (°)	28.45 ± 9.01	25.48 ± 8.91	29.89 ± 8.72	**<0.001**
L1-NB (°)	29.80 ± 6.76	28.46 ± 6.60	30.44 ± 6.76	**0.035**
ANB (°)	4.05 ± 2.51	4.17 ± 2.49	3.99 ± 2.53	0.605
SNA (°)	81.58 ± 3.75	81.29 ± 3.97	81.72 ± 3.65	0.428
SNB (°)	77.23 ± 5.97	77.10 ± 3.87	77.29 ± 6.77	0.789
Curve of Spee (mm)	2.97 ± 1.26	2.88 ± 1.15	3.01 ± 1.32	0.441
MP-FH (°)	26.69 ± 7.06	26.97 ± 6.01	26.56 ± 7.53	0.656

**Table 4:** Performance comparison of six machine learning models on the extraction test set.

Model	AUC (95% CI)	F1 (95% CI)	PR-AUC (95% CI)	Precision (95% CI)	Recall (95% CI)
AHP+RF	0.864 (0.791–0.913)	0.782 (0.701–0.850)	0.845 (0.787–0.923)	0.765 (0.701–0.891)	0.800 (0.667–0.857)
Original RF	0.820 (0.766–0.901)	0.770 (0.688–0.840)	0.834 (0.742–0.902)	0.743 (0.639–0.833)	0.800 (0.667–0.857)
XGBoost	0.835 (0.744–0.889)	0.779 (0.678–0.833)	0.843 (0.697–0.884)	0.746 (0.657–0.855)	0.815 (0.662–0.865)
AHP+LR	0.806 (0.686–0.841)	0.731 (0.598–0.771)	0.842 (0.672–0.870)	0.710 (0.582–0.794)	0.754 (0.583–0.797)
SVM	0.754 (0.689–0.843)	0.681 (0.617–0.787)	0.775 (0.714–0.877)	0.644 (0.583–0.790)	0.723 (0.618–0.817)
GBDT	0.838 (0.732–0.875)	0.758 (0.661–0.820)	0.835 (0.704–0.880)	0.746 (0.611–0.808)	0.769 (0.677–0.869)

**Table 5:** Performance comparison of six machine learning models on the maximum anchorage test set.

Model	AUC (95% CI)	F1 (95% CI)	PR-AUC (95% CI)	Precision (95% CI)	Recall (95% CI)
AHP+RF	0.808 (0.683–0.929)	0.909 (0.825–0.948)	0.925 (0.882–0.984)	0.926 (0.754–0.939)	0.893 (0.865–1.000)
Original RF	0.637 (0.591–0.757)	0.862 (0.763–0.919)	0.901 (0.842–0.969)	0.870 (0.709–0.909)	0.855 (0.782–0.962)
XGBoost	0.731 (0.651–0.889)	0.866 (0.775–0.922)	0.914 (0.881–0.977)	0.870 (0.714–0.911)	0.862 (0.811–0.980)
AHP+LR	0.822 (0.721–0.931)	0.941 (0.872–0.976)	0.934 (0.903–0.986)	0.963 (0.936–0.989)	0.920 (0.761–0.947)
SVM	0.803 (0.617–0.906)	0.940 (0.907–0.974)	0.827 (0.804–0.879)	0.975 (0.877–0.987)	0.908 (0.875–0.941)
GBDT	0.715 (0.702–0.817)	0.877 (0.813–0.938)	0.922 (0.903–0.983)	0.889 (0.759–0.943)	0.865 (0.841–0.981)

**Table 6:** Expert Evaluation Results.

Evaluation Dimension	Average Score ± SD (n = 5)	Representative Expert Comments
Perceived rationality of recommendations	8.5 ± 0.6	The treatment plan formulation is clear and accurate
Perceived workflow efficiency	9.3 ± 0.5	Significantly shortens the time needed for treatment planning
Perceived interpretability	9.1 ± 0.4	SHAP value visualization is relatively user-friendly for doctors
Perceived potential clinical value	8.8 ± 0.7	Suitable for assisting doctors’ judgment and teaching

## Data Availability

The clinical datasets used and/or analyzed during the current study, including the internal development dataset and the independent external validation cohort, are not publicly available due to institutional and ethical restrictions but may be made available from the corresponding author upon reasonable request and subject to institutional approval. The complete analysis code used for data preprocessing, AHP score construction, fuzzy-rule scoring, model training, hyperparameter tuning, SHAP interpretation, calibration assessment, decision curve analysis, bootstrap confidence interval estimation, DeLong testing, and external validation has been deposited in Zenodo with a citable https://doi.org/10.5281/zenodo.20440406. The code is publicly available under the stated open-source license.

## Supplementary Materials

The following supporting information can be downloaded at: https://www.mdpi.com/article/10.3390/bioengineering13060671/s1, Figure S1: Calibration and decision curve analyses of the selected models in the external validation cohort. (a) Calibration curve for extraction prediction. (b) DCA for extraction prediction. (c) Calibration curve for maxi-mum-anchorage prediction. (d) DCA for maximum-anchorage prediction; Table S1: Inter-observer reliability of repeated cephalometric measurements; Table S2: Expert-derived fuzzy scoring rules for maximum-anchorage tendency; Table S3: Threshold-sensitivity analysis for the selected models; Table S4: (a) Ablation analysis of the AHP composite score for extraction prediction; (b) Ablation analysis of knowledge-informed features for maximum-anchorage prediction; Table S5: Paired DeLong tests for AUC comparisons of the selected models against alternative models; Table S6: Exploratory end-to-end performance of the deployable two-stage cascade in the external validation cohort; Table S7: External validation performance and calibration of the proposed models; Table S8: (a) Comparison of extraction-model predictors between the development and external validation cohorts; (b) Comparison of maximum-anchorage-model predictors between the development and external validation extraction cohorts.

## Abbreviations

AHP	Analytic H1
AHP+RF	Analytic Hierarchy Process–Random Forest
AHP+LR	Analytic Hierarchy Process–Logistic Regression
AI	Artificial intelligence
ANB	Point A–Nasion–Point B angle
ANS-Me	Anterior nasal spine to menton (distance)
APDI	Anteroposterior dysplasia indicator
AUC	Area under the ROC curve
PR-AUC	Precision–recall curve
CI	Confidence interval
CR	Consistency ratio
DCA	Decision curve analysis
GBDT	Gradient boosting decision tree
L1-NB	Lower incisor to NB angle
LR	Logistic regression
ML	Machine learning
MP-FH	Mandibular plane–Frankfort horizontal angle
RF	Random forest
ROC	Receiver operating characteristic
SHAP	SHapley Additive exPlanations
SNA	Sella–Nasion–Point A angle
SNB	Sella–Nasion–Point B angle
SVM	Support vector machine
U1-NA	Upper incisor to NA angle
XGBoost	Extreme Gradient Boosting
