# Supplementary material for: Development and External Validation of an Explainable AHP-ML Model for Orthodontic Tooth Extraction and Anchorage Decision Support
Source: Bioengineering (Basel). 2026 Jun 10;13(6):671. doi: 10.3390/bioengineering13060671 (PMC13295870; doi:10.3390/bioengineering13060671)
Supplement: Supplementary file 1 [file bioengineering-13-00671-s001.zip › bioengineering-4316008-supplementary.pdf]

Supplementary Materials

Table S1. Inter-observer reliability of repeated cephalometric measurements

Variable	n	ICC(A,1)	Dahlberg error
SNA	20	0.907	0.725°
SNB	20	0.971	0.579°
ANB	20	0.984	0.389°
U1–NA	20	0.947	1.974°
L1–NB	20	0.970	1.014°
MP–FH	20	0.981	0.918°
Nasolabial angle	20	0.998	0.187°
Lower lip to E-line distance	20	0.959	0.438 mm

Table S2. Expert-derived fuzzy scoring rules for maximum-anchorage tendency.

Indicator	Low tendency = 0	Moderate tendency = 0.5	High tendency = 1
U1-NA	≤ 25°	> 25° and ≤ 30°	> 30°
Lower lip to E-line distance	≤ 2 mm	> 2 mm and ≤ 4 mm	> 4 mm
Upper arch crowding	≤ 3 mm	> 3 mm and ≤ 6 mm	> 6 mm
Lower arch crowding	≤ 3 mm	> 3 mm and ≤ 6 mm	> 6 mm
L1-NB	≤ 25°	> 25° and ≤ 30°	> 30°
Overjet	Normal / mild / reverse overjet	Moderate	Severe
Molar relationship	Class III	Class II	Class I
Overbite	Normal / mild / anterior open bite	Moderate	Severe
ANS-Me	≤ 65 mm	> 65 mm and ≤ 70 mm	> 70 mm

Table S3. Threshold-sensitivity analysis for the selected models.

Prediction task	Threshold method	Threshold	Recall	Specificity	F1	Net benefit
Extraction	Prespecified threshold	0.500	0.800	0.809	0.782	0.287
	Youden index	0.467	0.809	0.779	0.797	0.308
	Maximum net benefit	0.203	0.971	0.441	0.767	0.414
Maximum anchorage	Prespecified threshold	0.500	0.920	0.901	0.941	0.554
	Youden index	0.729	0.924	0.957	0.948	0.564

	Treat-all asymptote / maximum apparent net benefit	0.200	0.980	0.000	0.770	0.615

Table S4a. Ablation analysis of the AHP composite score for extraction prediction.

**Model**	**AUC**	**PR-AUC**	**F1**	**Precision**	**Recall**	**Brier**
Clinical RF	0.820	0.834	0.770	0.743	0.800	0.175
Proposed AHP+RF	0.864	0.845	0.782	0.765	0.800	0.171

Table S4b. Ablation analysis of knowledge-informed features for maximum-anchorage prediction.

**Model**	**AUC**	**PR-AUC**	**F1**	**Precision**	**Recall**	**Brier**
Clinical LR	0.806	0.931	0.927	0.914	0.940	0.172
Clinical + AHP LR	0.818	0.940	0.916	0.900	0.933	0.156
Clinical + Expert LR	0.808	0.941	0.935	0.937	0.933	0.170
Proposed AHP+LR	0.822	0.934	0.941	0.963	0.920	0.153

Table S5. Paired DeLong tests for AUC comparisons of the selected models against alternative models

**Prediction task**	**Comparison**	**AUC difference**	**P value**
Extraction	AHP+RF vs. Original RF	0.044	0.016
	AHP+RF vs. XGBoost	0.029	0.062
	AHP+RF vs. AHP+LR	0.058	0.006
	AHP+RF vs. SVM	0.110	0.003
	AHP+RF vs. GBDT	0.026	0.053
Maximum anchorage	AHP+LR vs. AHP+RF	0.014	0.081
	AHP+LR vs. Original RF	0.185	0.001
	AHP+LR vs. XGBoost	0.091	0.016
	AHP+LR vs. SVM	0.019	0.073
	AHP+LR vs. GBDT	0.107	0.003

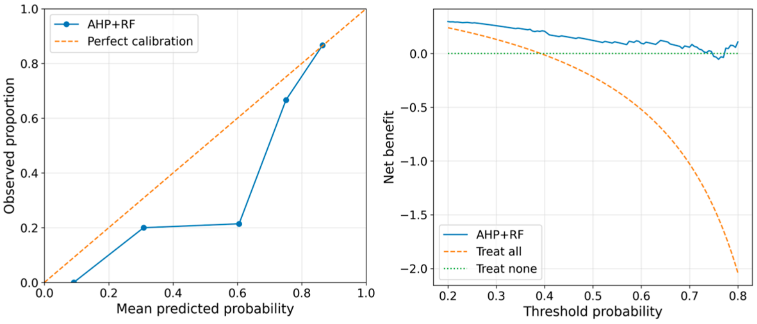

(a) (b)

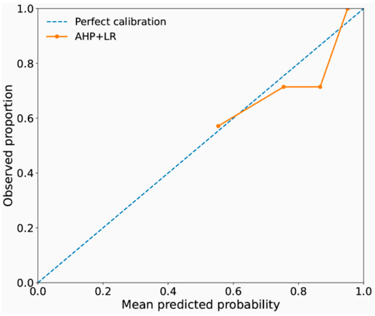

(c)

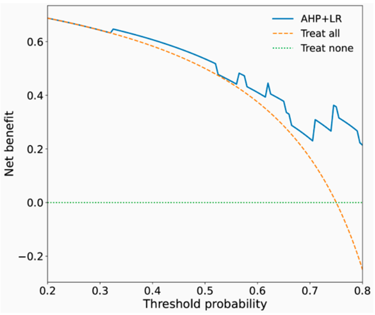

(d)

Figure S1. Calibration and decision curve analyses of the selected models in the external validation cohort. (a) Calibration curve for extraction prediction. (b) DCA for extraction prediction. (c) Calibration curve for maximum-anchorage prediction. (d) DCA for maximum-anchorage prediction.

Table S6. Exploratory end-to-end performance of the deployable two-stage cascade in the external validation cohort.

Item	Value
Total external validation cases	74
Patients entering the second-stage anchorage model after first-stage prediction	40
True extraction cases missed by the first-stage model	3
Incorrect anchorage decisions after entering the second stage	3
Final downstream error count among true extraction cases	6

Table S7. External validation performance and calibration of the proposed models.

Task	Metric	Point estimate	95% CI
Tooth extraction prediction	Accuracy	77.03%	66.25%-85.13%
	Sensitivity	89.66%	73.61%-96.42%
	Specificity	68.89%	54.34%-80.47%
	PPV	65.00%	49.51%-77.87%
	NPV	91.18%	77.04%-96.95%
	AUC	0.79	0.68–0.90
	Brier score	0.210	-
	Calibration intercept	-0.271	-
Maximum-anchorage prediction	Accuracy	82.76%	65.45%-92.40%
	Sensitivity	90.91%	72.19%-97.47%
	Specificity	57.14%	25.05%-84.18%
	PPV	86.96%	67.87%-95.46%
	NPV	66.67%	30.00%-90.32%
	AUC	0.82	0.66–0.98
	Brier score	0.170	-
	Calibration intercept	-0.316	-

Table S8a. Comparison of extraction-model predictors between the development and external validation cohorts.

Variable	Development cohort (n = 485)	External validation cohort(n = 74)	P value
Lower lip to E-line distance	1.95 ± 3.10	3.36 ± 4.54	0.012
Nasolabial angle	102.96 ± 10.03	100.06 ± 9.77	0.020
Upper arch crowding	3.25 ± 3.68	3.68 ± 4.04	0.391
Lower arch crowding	2.73 ± 3.26	3.43 ± 3.40	0.101
U1-NA	25.97 ± 9.14	27.32 ± 8.92	0.230
L1-NB	27.66 ± 6.89	24.19 ± 7.12	<0.001
ANB	3.87 ± 2.65	3.34 ± 2.45	0.090
SNA	81.76 ± 3.73	81.74 ± 3.73	0.966
SNB	77.76 ± 5.12	78.40 ± 3.89	0.211
Curve of Spee	2.88 ± 1.23	2.70 ± 1.09	0.197
MP-FH	25.83 ± 6.63	27.79 ± 6.32	0.015
Extraction	239/485 (49.3%)	29/74 (39.2%)	0.106

Table S8b. Comparison of maximum-anchorage-model predictors between the development and external validation extraction cohorts.

Variable	Development extraction cohort (n = 239)	External validation extraction cohort (n = 29)	P value
Molar relationship			0.011
Class I	122/239 (51.0%)	11/29 (37.9%)	
Class II	77/239 (32.2%)	17/29 (58.6%)	
Class III	40/239 (16.7%)	1/29 (3.4%)	
Overbite			0.384
Normal	93/239 (38.9%)	15/29 (51.7%)	
Mild	29/239 (12.1%)	3/29 (10.3%)	
Moderate	54/239 (22.6%)	7/29 (24.1%)	
Severe	39/239 (16.3%)	4/29 (13.8%)	
Anterior open bite	24/239 (10.0%)	0/29 (0.0%)	
Overjet			0.105
Normal	98/239 (41.0%)	17/29 (58.6%)	
Mild	46/239 (19.2%)	2/29 (6.9%)	
Moderate	42/239 (17.6%)	7/29 (24.1%)	
Severe	29/239 (12.1%)	3/29 (10.3%)	
Reverse overjet	24/239 (10.0%)	0/29 (0.0%)	
Upper arch crowding	4.08 ± 4.06	6.81 ± 4.86	0.007
Lower arch crowding	3.34 ± 3.42	5.57 ± 3.20	0.001
U1-NA	28.45 ± 9.01	27.30 ± 7.41	0.446
L1-NB	29.80 ± 6.76	26.91 ± 6.37	0.028
Lower lip to E-line distance	3.00 ± 3.24	5.61 ± 4.28	0.003
ANS-Me	66.82 ± 6.79	66.19 ±5.34	0.565
Maximum anchorage	152/239 (63.6%)	22/29 (75.9%)	0.191
